# Whole genome sequencing and analysis of selenite-reducing bacteria *Bacillus paralicheniformis* SR14 in response to different sugar supplements

**DOI:** 10.1186/s13568-023-01598-9

**Published:** 2023-09-04

**Authors:** Fengqin Wang, Tao Gong, Man Du, Xiao Xiao, Zipeng Jiang, Weilian Hu, Yizhen Wang, Yuanzhi Cheng

**Affiliations:** 1 Key Laboratory of Molecular Animal Nutrition, Ministry of Education, Hangzhou, 310058 China; 2 Key Laboratory of Animal Nutrition and Feed Science (Eastern of China), Ministry of Agriculture and Rural Affairs, Hangzhou, 310058 China; 3 School of Biological and Chemical Engineering, Zhejiang University of Science and Technology, Hangzhou, 310035 China

**Keywords:** *Bacillus paralicheniformis*, Selenite reduction, Whole genome sequence, Sugar supplements

## Abstract

The biosynthetic process of selenium nanoparticles (SeNPs) by specific bacterial strain, whose growth directly affects the synthesis efficiency, has attracted great attentions. We previously reported that *Bacillus paralicheniformis* SR14, a SeNPs-producing bacteria, could improve intestinal antioxidative function in vitro. To further analyze the biological characteristics of SR14, whole genome sequencing was used to reveal the genetic characteristics in selenite reduction and sugar utilization. The results reviewed that the genome size of SR14 was 4,448,062 bp, with a GC content of 45.95%. A total of 4300 genes into 49 biological pathways was annotated to the KEGG database. EC: 1.1.1.49 (glucose-6-phosphate 1-dehydrogenase) and EC: 5.3.1.9 (glucose-6-phosphate isomerase), were found to play a potential role in glucose degradation and EC:2.7.1.4 (fructokinase) might be involved in the fructose metabolism. Growth profile and selenite-reducing ability of SR14 under different sugar supplements were determined and the results reviewed that glucose had a better promoting effect on the reduction of selenite and growth of bacteria than fructose, sucrose, and maltose. Moreover, RT-qPCR experiment proved that glucose supplement remarkably promoted the expressions of thioredoxin, fumarate reductase, and the glutathione peroxidase in SR14. Analysis of mRNA expression showed levels of glucose-6-phosphate dehydrogenase and fructokinase significantly upregulated under the supplement of glucose. Overall, our data demonstrated the genomic characteristics of SR14 and preliminarily determined that glucose supplement was most beneficial for strain growth and SeNPs synthesis.

**Supplementary Information:**

The online version contains supplementary material available at 10.1186/s13568-023-01598-9.

## Keypoints

1. Whole genome sequencing was used to reveal the genetic characteristics of SR14 in selenite reduction and sugar utilization.
2. Supplementing glucose had the best promoting effect on growth and selenite reduction of SR14, and the effect was better than supplementing sucrose, fructose, or maltose.
3. EC:1.1.1.49 (glucose-6-phosphate 1-dehydrogenase) and EC:2.7.1.4 (fructokinase) might be associated with the metabolic process of SR14.
4. SR14 might rely on multiple enzymes, such as thioredoxin, fumarate reductase, and glutathione peroxidase, to reduce selenite when supplementing with glucose.

**Supplementary Information:**

The online version contains supplementary material available at 10.1186/s13568-023-01598-9.

## Introduction

Selenium is an essential trace element that incorporates into selenoproteins as selenocysteine representing the most important part of their active center (Khurana et al. 2019). Selenium has a narrow threshold between therapy and toxicity, whereas the selenium nanoparticles (SeNPs) possess remarkably lower toxicity than selenate, selenite and organic selenium (Wang et al. 2007). Several investigations have demonstrated that biogenic SeNPs have antimicrobial (Vaquette et al. 2020), antioxidant (Ge et al. 2022), anticancer (Wang et al. 2022a) and hormone secretion promoting properties (Ojeda et al. 2022). SeNPs-producing microorganism has attracted increasing attention in the recent years. *Bacillus* species, with strong environmental adaptability and prebiotic function, have become the suitable bioreactor to synthesize SeNPs (Ashengroph and Hosseini 2021; Hashem et al. 2021; Wu et al. 2016).

The residue of selenite and the content of SeNPs in culture medium are closely associated with microbial activity, including growth rate, viable count and metabolism-related enzyme expression levels. Different type of sugar supplements played a crucial role in bacterial growth and high-value-added product biosynthesis (Hammi et al. 2016; Wang et al. 2018a). Guo et al. devised a new strategy to produce the isomaltulose by *Corynebacterium glutamicum* IS7, where the sucrose component was used as the substrate and the monosaccharides (glucose and fructose) were used as the energy source for strain growth (Guo et al. 2022). Meanwhile, current findings suggest that various enzymes in bacteria have been involved in SeNPs synthesis. Under aerobic conditions, enzymes such as glutathione reductase (Wadhwani et al. 2016), thioredoxin reductase (TrxR) (Hunter 2014), fumarate reductase (Song et al. 2017), and sulfite reductase (Wang et al. 2022b) have so far been reported to participate in the selenite reduction.

Whole genome sequencing is now available for many microbial species to assess their safety traits (Salvetti et al. 2016), evolution traits (Zhang et al. 2016), and metabolic function (Xu et al. 2017). Jia et al. preliminary revealed that the reduction of selenite by *Bacillus subtilis* 168 was mediated by multiple pathways both in vivo and in vitro (Jia et al. 2022). Nevertheless, the mechanism of selenite reduction under different fermentation conditions was not elucidated.

*B. paralicheniformis* SR14, a strain that we isolated before, possessed excellent selenite resistance and SeNPs producing ability (Cheng et al. 2017; Xiao et al. 2019). The present study aimed to explore the genes related to selenite reduction and sugar utilization in SR14 by whole genome sequencing analysis. Herein, we (i) annotated the predicted genes using Kyoto Encyclopedia of Genes and Genomes (KEGG) databases; (ii) characterized the difference in the selenite reducing ability under different sugar supplements; and (iii) verified the potential functional genes by RT-qPCR. Hence, it is important to determine the optimal sugar supplement for the growth of the strain to improve the SeNPs synthesize ability and reveal the potential enzymes in specific bacteria strain.

## Materials and methods

### Bacterial strain and chemicals

*B. paralicheniformis* SR14, a selenite-tolerated bacterial strain (CGMCC No. 13908), was identified and kept in our laboratory. Yeast extract and tryptone were purchased from OXOID (Hampshire, UK). Phosphate buffer powder (PBS), glucose, fructose, sucrose, maltose, NaCl, K_2_HPO_4_, and MgSO_4_ were purchased from Sinopharm Chemical Reagent Co., Ltd. (Shanghai, China). Sodium selenite (CAS: 10,102-18-8) was purchased from Sigma-Aldrich (St. Louis, MO, USA). TRIzol reagent was purchased from Invitrogen (Waltham, MA, USA).

### Microorganism cultivation and determination of selenite contents

The cultivation process of SR14 was designed according to the method we described before with minor modifications (Cheng et al. 2017). Briefly, a single colony of SR14 was initially cultured in LB medium at 37 °C overnight. Next, 500 μL of the seed fluid was inoculated into 50 mL of different cultivation culture groups. The minimum broth medium was used as the negative control (NC). Detailed grouping information was listed in Table 1.

**Table 1:** Group assignment

Assignment	Abbreviation	Component	
		Common components	Different sugar sources
Control group	Con	10 g/L tryptone + 10 g/L yeast extract + 1 g/L K_2_HPO_4_ + 5 g/L NaCl + 1.5 g/L MgSO_4_	–
Glucose group	Glu		20 g/L glucose
Fructose group	Fru		20 g/L fructose
Sucrose group	Suc		20 g/L sucrose
Maltose group	Mal		20 g/L maltose

### Whole genome sequencing, assembly, and annotation

Cultures of SR14 in grown to the exponential phase without the presence of selenite was collected and centrifuged at 4 °C at 5000 ×*g* for 5 min. The genomic DNA was extracted, qualified, and determined. The whole genome shotgun strategy was used to build libraries with different inserted fragments at Personalbio Co., Ltd (Shanghai, China). The second-generation sequencing was performed on the Illumina Novaseq 6000 platform. The third-generation sequencing was performed on the Oxford Nanopore ONT. FastQC strategy was used to control the quality of data (Patel and Jain 2012).

### Selenite reducing capacity and strain growth ability

The effect of different kinds of sugar supplement on the growth of SR14 was determined without the presence of sodium selenite. The remaining levels of selenite were determined using ICP-MS (Thermo Fischer Scientific, Waltham, MA, USA). Briefly, the culture medium was filtered through a 0.22 µm aqueous nylon flter membrane. Then the signal intensity of ^78^Se was subsequently determined analytically in KED mode and compared with the sodium selenite standard solution.

### RT-PCR assay

RNA samples of SR14 from different groups were extracted using TRIzol reagent. The quantity and quality of RNA were determined by NanoDrop 2000 spectrophotometer (Thermo Fisher Scientific, MA, USA). After examination of RNA purity and concentration, 2 μg of RNA was used as a template to reverse transcribe to cDNA by using M-MLV Reverse Transcriptase Kit. qPCR analysis was performed using the SYBR Green PCR Master Mix with the ABI Step-One PlusTM Real-Time PCR System (Applied Biosystems, Waltham, MA, USA). The mRNA relative expression was calculated using the 2^−ΔΔCt^ method. Primers were listed in Table 2.

**Table 2:** Primer sequences for RT-PCR

Gene	Sequence (5′ → 3′)
Fumarate hydratase	Forward: AAAATCGGCCGCACTCATTT Reverse: GATGGCAAGATCGCGGATTT
Glucose-6-phosphate dehydrogenase	Forward: CAGGGGATTTGGCAAAACGA Reverse: TCGAAACCGATTGCTGTACG
Glutathione peroxidase	Forward: ATCAAATCCACCCGCTGTTC Reverse: TGGATTGGTTTGCGGTGAAA
Maltose-6′-phosphate glucosidase	Forward: CCCGGGGATTGTATTGATGC Reverse: GAAAACGCCTCTTCCGGATC
Thioredoxin reductase	Forward: GCCGGTACTTGAAGAGCTTG Reverse: GCCGACAGATGTTTCAACCA
Fructokinase	Forward: CAGTCGGTACAGGAATCGGA Reverse: CCCGATGCCATTCCTTCAAG
16S	Forward: ACTCCTACGGGAGGCAGCA Reverse: GGACTACHVGGGTWTCTAAT

### Statistical analysis

All data were expressed as means ± SD. One-way analysis of variance (ANOVA) followed by a Tukey multiple comparison test was used to determine the statistical significance for multiple comparisons. Differences between groups were considered statistically significant at *P < 0.05.

## Results

### Whole genome sequencing and assembly of *B. paralicheniformis* SR14

Whole-genome sequencing using Illumina NovaSeq and Oxford Nanopore ONT was established to generate insight into the mechanism of selenite reduction by SR14. As shown in Table 3, a total length of 4,448,062 bp, with a GC content of 45.95%, were predicted in the genome.

**Table 3:** Genome statistics of *Bacillus paralicheniformis* SR14

Attributes	Values
Length (bp)	4,448,062
GC content (%)	45.95
Sequence type	circular
ORF numbers	4560
ORF total length (bp)	3,902,469
ORF/Genome (coding percentage, %)	87.73
tRNA numbers	81
rRNA numbers	24
ncRNA numbers	105
CRISPR repeats	2

**Fig. 1:**
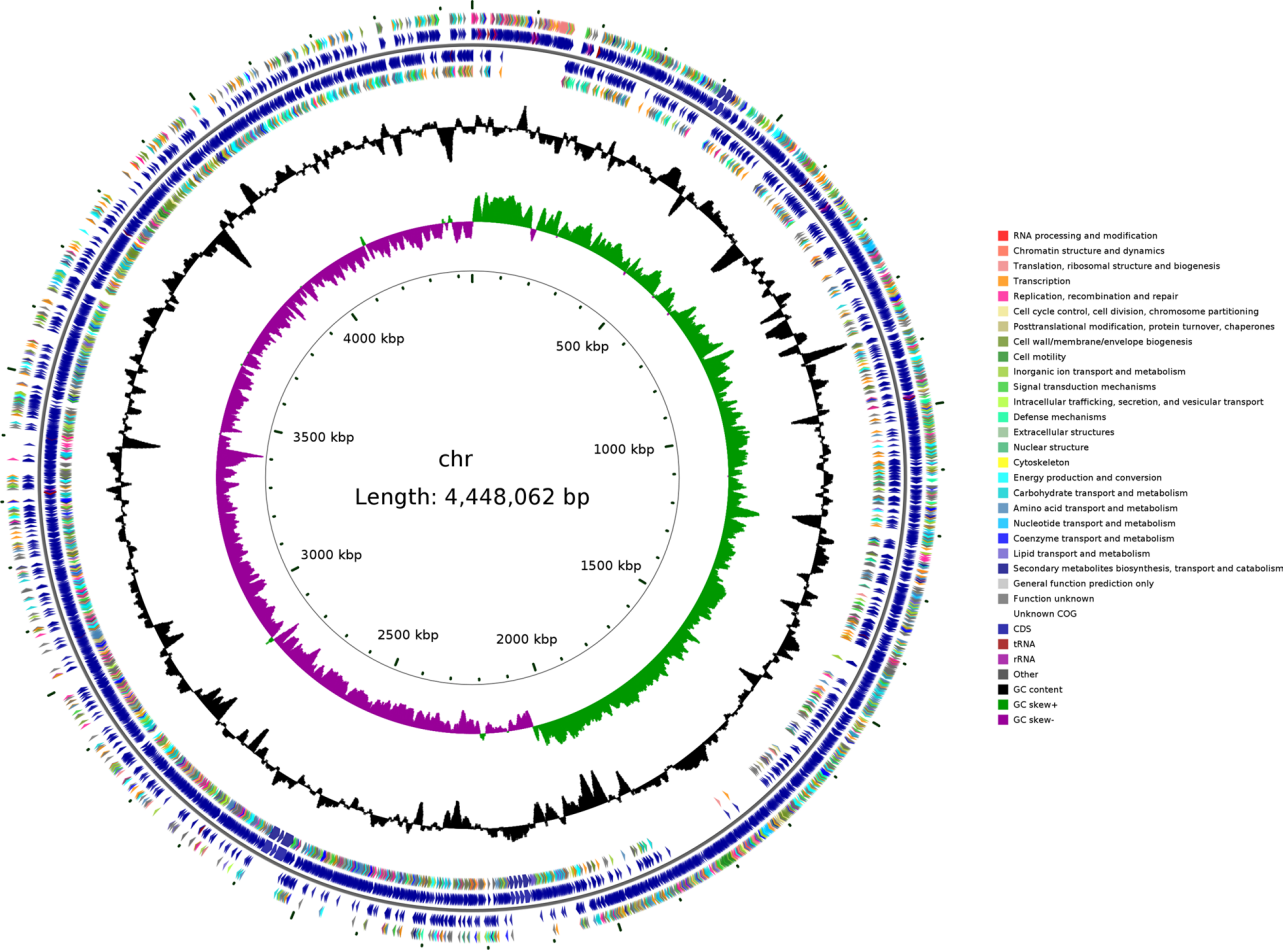
Circular map of *B. paralicheniformis* SR14 genome. From the center to outside are the scale, GC skew, GC content, the COG of each CDS, the positions of CDS, tRNA, and rRNA, respectively

We integrated the genome sequence, gene prediction and non-coding RNA prediction information into standard GenBank format file. As detailed in Fig. 1, the results indicated that a circular plot of the genome, including the number of bases, GC content, GC skew, and location of all annotated open reading frames (ORFs) sorted by the clusters of orthologous gene (COG) category and colored.

### Analysis of sugar utilization and selenite reducing characteristics in genome of *B. paralicheniformis* SR14

**Fig. 2:**
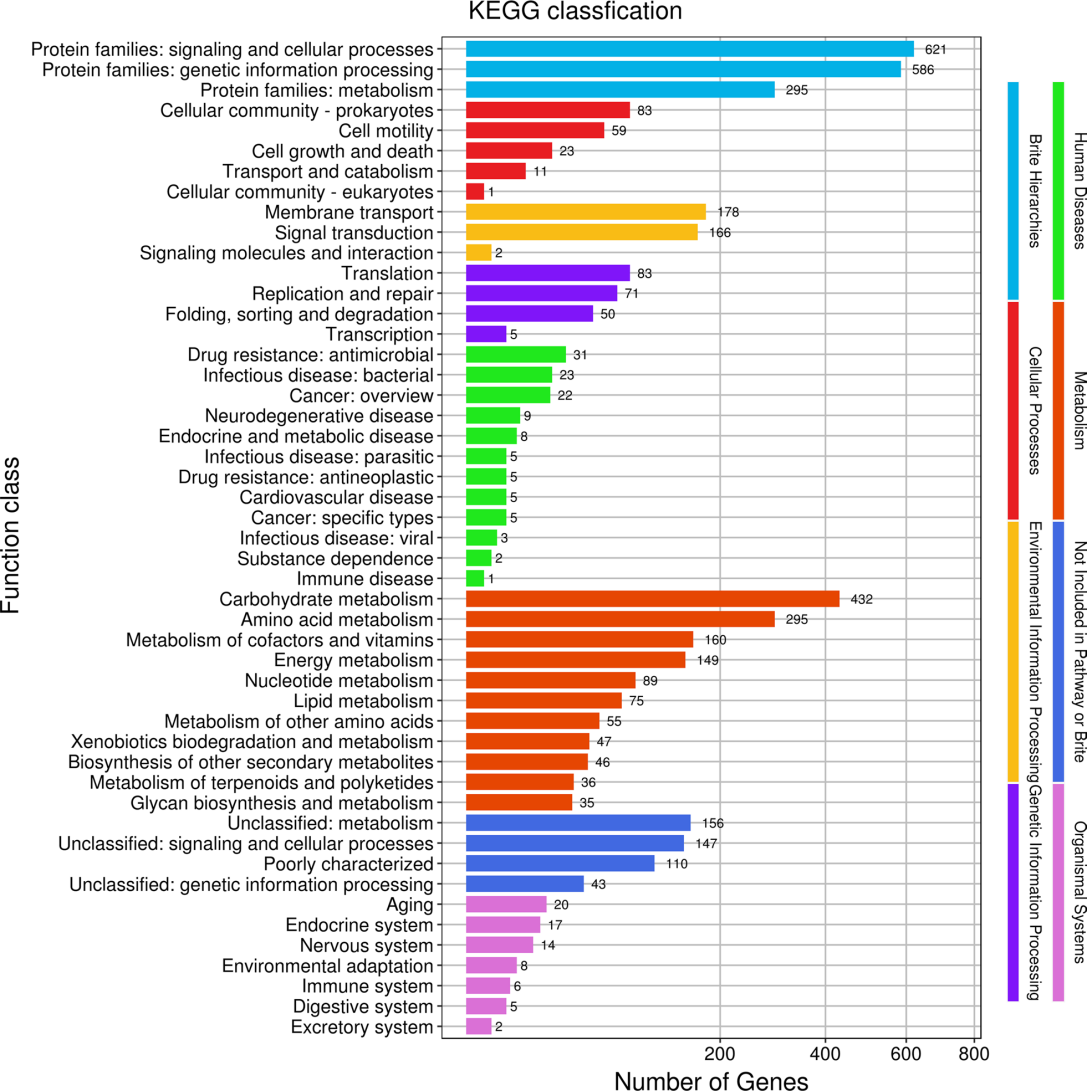
Circular map of *B. paralicheniformis* SR14 genome. From the center to outside are the scale, GC skew, GC content, the COG of each CDS, the positions of CDS, tRNA, and rRNA, respectively

To better comprehend the sugar and selenite metabolic pathways of SR14, we further annotated a total of 4300 genes into 49 biological pathways in the KEGG database (Fig. 2), of which 432 genes were assorted as carbohydrate metabolism genes, and 149 genes were assorted as energy metabolism genes.

Genes associated with the specific metabolic process were crucial for biological process category. There were 179 genes annotated with sugar metabolism in the SR14 genome, among which 47 were concerned with the maltose metabolism, 69 were concerned with fructose metabolism, 60 genes were associated with sucrose metabolism, and 3 genes were associated with maltose metabolism. As shown in Additional file 1: Fig. S1a, pentose phosphate pathway related genes, such as EC: 1.1.1.49 (glucose-6-phosphate 1-dehydrogenase) and EC: 5.3.1.9 (glucose-6-phosphate isomerase), were found to play a potential role in glucose degradation. Meanwhile, EC: 2.7.1.4 (fructokinase) might be involved in the fructose metabolism **(**Additional file 1: Fig. S1b and S1c). Subsequently, glucose, fructose, sucrose, and maltose were selected for the growth profile and selenite-reducing experiment.

**Fig. 3:**
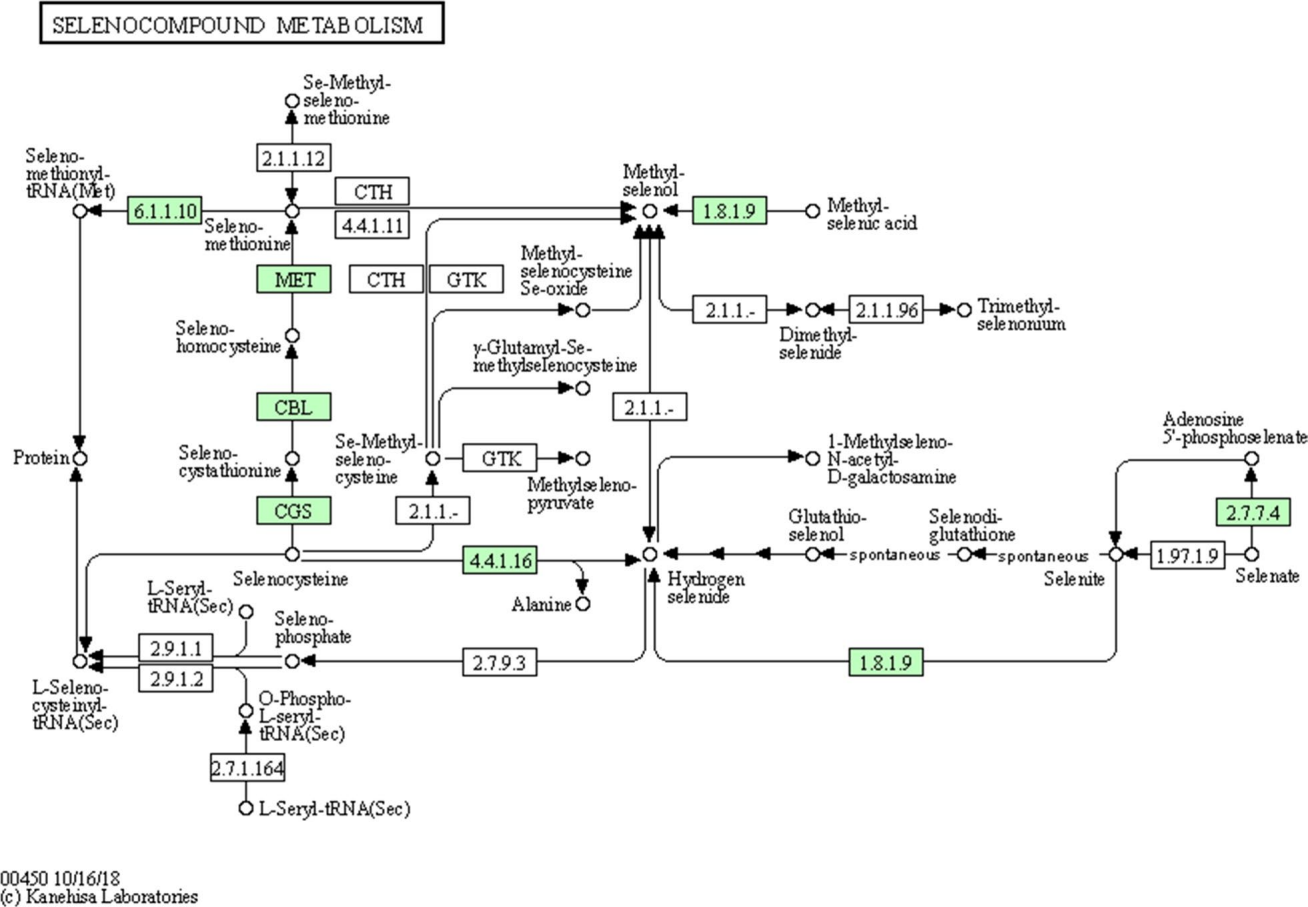
Potential pathway of selenocompound metabolism process. The green boxes represented the key genes contained in SR14

Furthermore, a total of 8 genes possibly played a role in the selenocompound metabolism process, such as EC: 6.1.1.10 (methionyl-tRNA synthetase) and EC: 1.8.1.9 (TrxR), were further screened (Fig. 3).

### Growth profile and selenite-reducing ability of *B. paralicheniformis* SR14 under different sugar supplement conditions

**Fig. 4:**
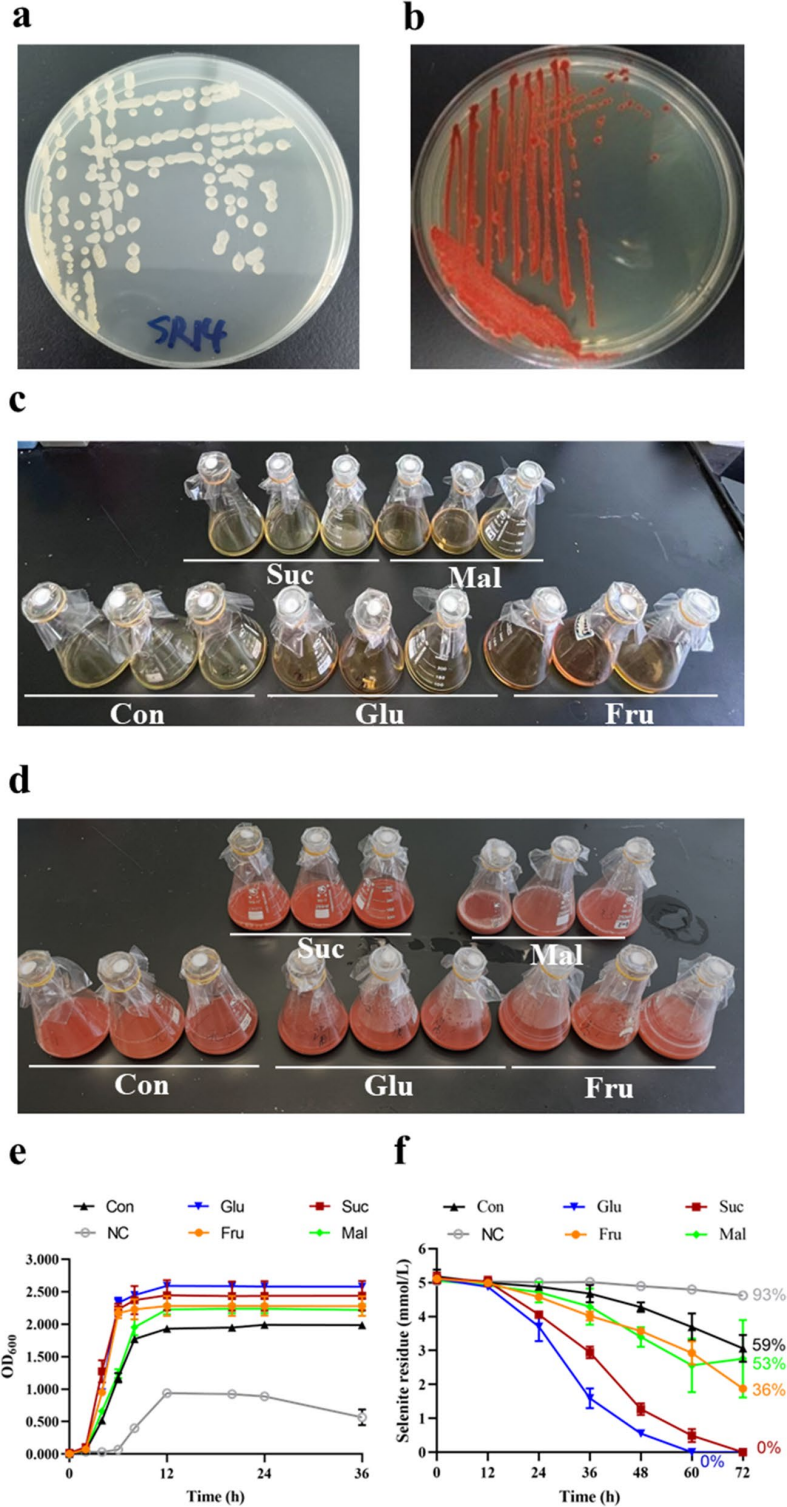
Growth and selenite reduction analysis of SR14. **a** image of SR14 colony on LB medium plate without selenite. **b** image of SR14 colony on LB medium plate with 2 mM selenite. **c** images of five different kinds of fluid medium before fermentation. **d** images of five different kinds of fluid medium after fermentation. **e** growth curves of SR14 in different mediums. **f** selenite reduction results SR14 in different mediums (n = 3)

As shown in Fig. 4b, the colonies of SR14 turned red with the addition of sodium selenite, suggesting that SR14 exhibited selenite tolerance and elemental selenium reduction abilities. After cultivating with the presence of 5 mg/L sodium selenite, the color of broth in five different groups was all changed to red (Fig. 4c and d).

The effect of different kinds of sugar on the growth of SR14 was determined. The results revealed that sugar supplement promoted the growth of SR14, with glucose being used for optimal choose (Fig. 4e). ICP-MS analysis indicated that in the Glu group and Suc group, the sodium selenite was totally reduced at 60 h and 72 h after inoculation, respectively. Nevertheless, even after 72 h of fermentation, there was still residual sodium selenite (93% for NC group; 59% for Con group; 53% for Mal group; and 36% for Fru group) in the broth of other groups (Fig. 4f).

### Analysis of the mRNA abundance of selected genes under different sugar supplementary

**Fig. 5:**
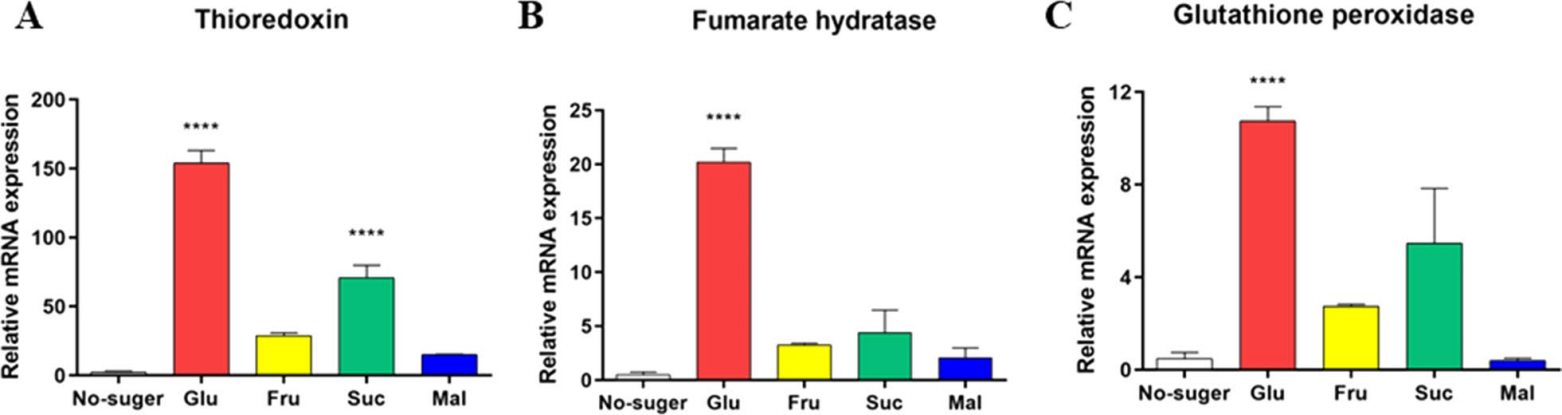
Relative expression of selenite-reducing genes in SR14. Glu: glucose group; Fru: fructose group; Suc: sucrose group; Mal: maltose group. *P < 0.05, **P < 0.01 (n = 3)

To verify the relative expression of selenite-reducing genes revealed in the genome-seq results, the mRNA abundance of the selenite reductase, including TrxR, fumarate reductase, and glutathione peroxidase, was assessed. As shown in Fig. 5, glucose supplement significantly upregulated the expressions of TrxR, fumarate reductase, and the glutathione peroxidase by 153.9-fold (P < 0.0001), 20.2-fold (P < 0.0001), and 10.7-fold (P < 0.001). Furthermore, sucrose supplement upregulated the expressions of TrxR by 70.7-fold (P < 0.0001).

**Fig. 6:**
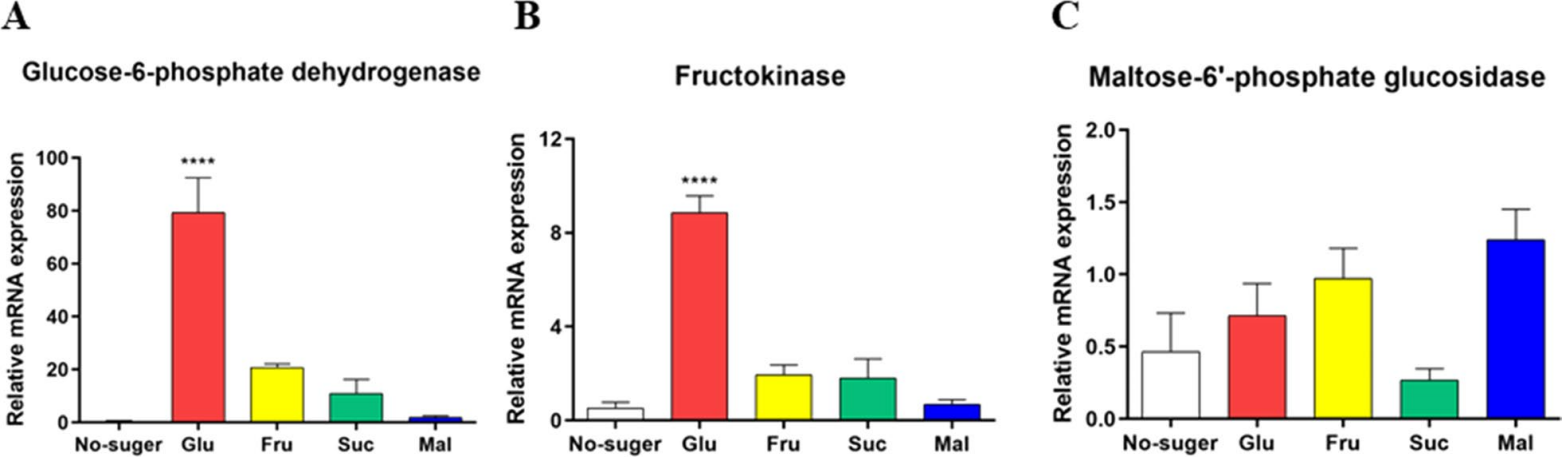
Relative expression of sugar metabolism genes in SR14. Glu: glucose group; Fru: fructose group; Suc: sucrose group; Mal: maltose group. *P < 0.05, **P < 0.01 (n = 3)

As shown in Fig. 6, the mRNA expression of glucose-6-phosphate dehydrogenase were significantly upregulated under the supplement of glucose by comparison with three other groups (79.3-fold). Meanwhile, glucose supplement significantly increased the mRNA expression of fructokinase. Nevertheless, all of four kind of sugar supplements did not significantly affect the expression of maltose-6′-phosphate glucosidase.

## Discussion

*B. paralicheniformis* SR14, a selenite-tolerated and polysaccharide-producing strain, exhibited great antioxidant properties in vitro (Cheng et al. 2017; Xiao et al. 2019). To further investigate the possible mechanism on its selenite reduction, the genome-wide analysis was used in this study. Our previous study had shown the genomic characteristics and antibacterial capacity of *Bacillus amyloliquefaciens* BA40 against *Clostridium perfringens* (Jiang et al. 2022). In this study, our analyses indicated that the expression of sugar metabolism, energy metabolism, and selenoprotein metabolism pathway genes played an important role in SR14 fermentation. The results showed that SR14 possessed an abundance of specific genes. Fructokinase, a key enzyme involved in the process of carbohydrate degradation of *Bacillus subtilis* (Nocek et al. 2011), *Corynebacterium glutamicum* (Peng et al. 2011), and *Zobellia galactanivorans* (Groisillier et al. 2015), turned out to be significantly upregulated in glucose-supplement group of SR14. In addition, glucose-6-phosphate 1-dehydrogenase participated in in NADPH-related glucose metabolism in prokaryotes, such as *Escherichia coli* (Xia et al. 2015), *Mycolicibacterium neoaurum* (Tang et al. 2021), and *Cyanobacteria* (Tiruveedula and Wangikar 2017). Glucose-6-phosphate 1-dehydrogenase and fructokinase mutant strain of SR14 would be constructed for further experiment.

The selection of different sugar supplements is a major factor influencing the growth of bacteria, thereby affecting their sodium selenite reduction ability. Glucose and sucrose have been shown to support the strain to detoxify selenite (Garbisu et al. 1995). Kashiwa et al. revealed that *Bacillus* sp. SF-1 was able to utilize a variety of organic acids or sugars, including acetate, citrate, fructose, glycerol, glucose, and sucrose, as the carbon source in selenate reduction (Kashiwa et al. 2000). Besides, varies sugar supplements could lead to differences in the metabolites of *Bacillus*. Lu et al. indicated that compared with arabinose and sorbitol, fructose most efficiently increased the concentration of the essential component amino acids in *Bacillus amyloliquefaciens* fmb-60 (Lu et al. 2016). Furthermore, bacterial exopolysaccharide associated carbohydrates such as galactose, glucose, mannose, and rhamnose played a significant role in synthesis and stabilization of the SeNPs (Ghosh et al. 2022). In this study, SR14 exhibited the optimum growth and selenium reduction ability under glucose addition. Without sugar supplement, SR14 could reduce 1.94 ± 0.39 mM sodium selenite, which was consistent with reported study (Barlow et al. 2017). Moreover, SR14 could only reduce 0.42 ± 0.09 mM sodium selenite when there was no sugar in the culture medium.

Numerous theories existed regarding the process of bio-reduction of selenite to elemental selenium, which involved in multiple metabolic pathways (Wang et al. 2022a, b), including glutathione reductase-dependent reduction (Kessi and Hanselmann 2004), TrxR-dependent reduction (Rui et al. 2022), siderophore or sulfide-mediated reduction (Zannoni et al. 2008), and dissimilatory reduction (Song et al. 2017). In this study, RT-PCR results indicated that multiple selenite-reducing enzymes were found to be significantly expressed only after glucose supplementation, including thioredoxin, fumarate reductase, and glutathione peroxidase. TrxR is one of the most important selenoenzyme that would be mainly involved in the detoxification of selenite (Jia et al. 2022). A recent study showed that selenium-enriched *Bifidobacterium breve* YH68-Se enhanced activities of TrxR and glutathione peroxidase with increasing selenite concentration (Rui et al. 2022). Shimizu et al. showed that selenite was reduced by the thioredoxin system from *Pseudomonas stutzeri* (Shimizu et al. 2021), which was consistent with other studies on *Alcaligenes faecalis* (Wang et al. 2018b). Yasir et al. reveal that NAD(P)H-dependent TrxR was essential for selenite reduction in *Bacillus* sp. Y3 by proteomics analysis (Yasir et al. 2020). Fumarate reductase has been proven to be involved in nano-selenium synthesis (Oremland et al. 1999). Our previous research revealed that *E. cloacae* Z0206 reduced selenite using fumarate reductase, rather than thioredoxin (Song et al. 2017). We believed the selenite-reducing process of SR14 might be complex and relied on multiple different enzymes.

We further investigated the reasons why the growth and selenium reduction effect of SR14 after glucose supplementation was superior to other sugars. Based on the results of whole genome analysis, three representative sugar metabolism genes were selected and their mRNA expression was measured. The result showed that the expressions of glucose-6-phosphate 1-dehydrogenase, which could affect the intracellular NADH/NADPH ratio (Bao et al. 2015), and fructokinase, which phosphorylated d-fructose with ATP as a cofactor (Nocek et al. 2011), were remarkably upregulated in Glu group. These results might indicate that SR14 exhibited more vigorous metabolism under glucose supplementation conditions.

In conclusion, this work has identified that 179 genes annotated with sugar metabolism in the genome of SR14, among which 47 were concerned with the maltose metabolism, and 69 were concerned with fructose metabolism. We confirmed that supplementing glucose had the best promoting effect on growth and selenite reduction, which might be associated with glucose-6-phosphate 1-dehydrogenase. Meanwhile, SR14 might rely on thioredoxin, fumarate reductase, and glutathione peroxidase to reduce selenite.

### Supplementary Information

**Additional file 1: Figure S1. **Potential pathway of sugar metabolism process. **A** pentose phosphate pathway; **B** starch and sucrose metabolism; and **C** fructose and mannose metabolism. The green boxes represented the key genes contained in SR14. **Figure S2. **Potential pathway of glutathione metabolism process. The green boxes represented the key genes contained in SR14.

## Data Availability

The original contributions presented in the study are publicly available. This data can be found here: https://www.ncbi.nlm.nih.gov/bioproject/PRJNA944838.
