# Supplementary material for: Whole genome sequencing and analysis of selenite-reducing bacteria Bacillus paralicheniformis SR14 in response to different sugar supplements
Source: AMB Express. 2023 Sep 4;13:93. doi: 10.1186/s13568-023-01598-9 (PMC10477163; doi:10.1186/s13568-023-01598-9)
Supplement: Supplementary file 1 — Additional file 1: Figure S1. Potential pathway of sugar metabolism process. A pentose phosphate pathway; B starch and sucrose metabolism; and C fructose and mannose metabolism. The green boxes represented the key genes contained in SR14. Figure S2. Potential pathway of glutathione metabolism process. The green boxes represented the key genes contained in SR14. [file 13568_2023_1598_MOESM1_ESM.docx]

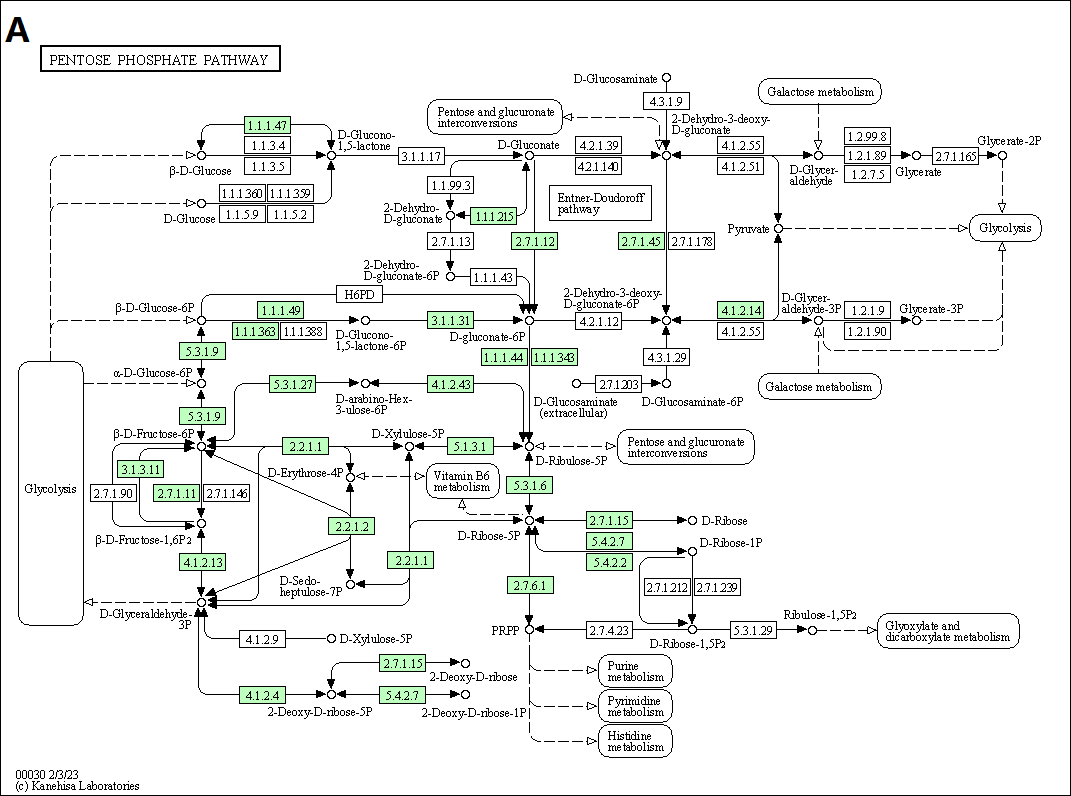

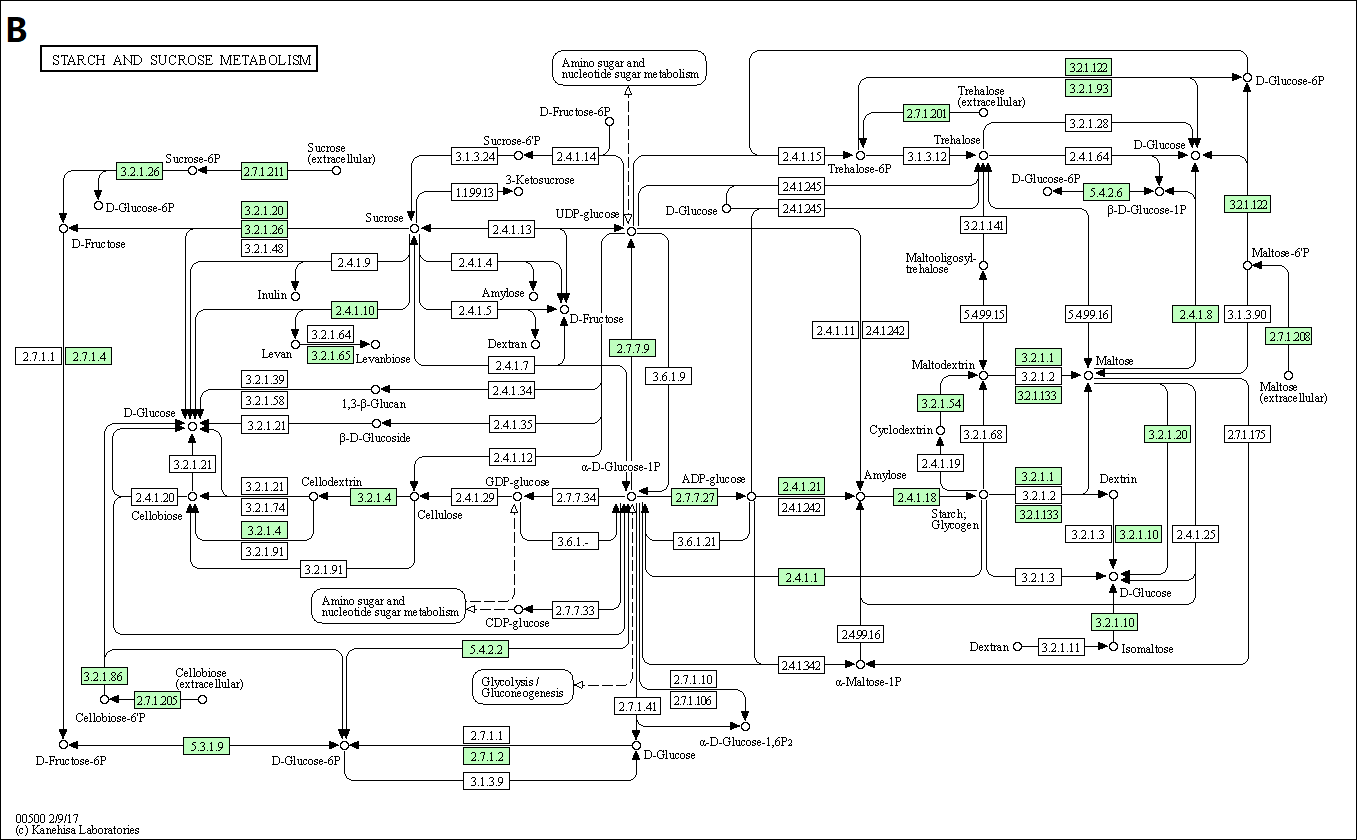

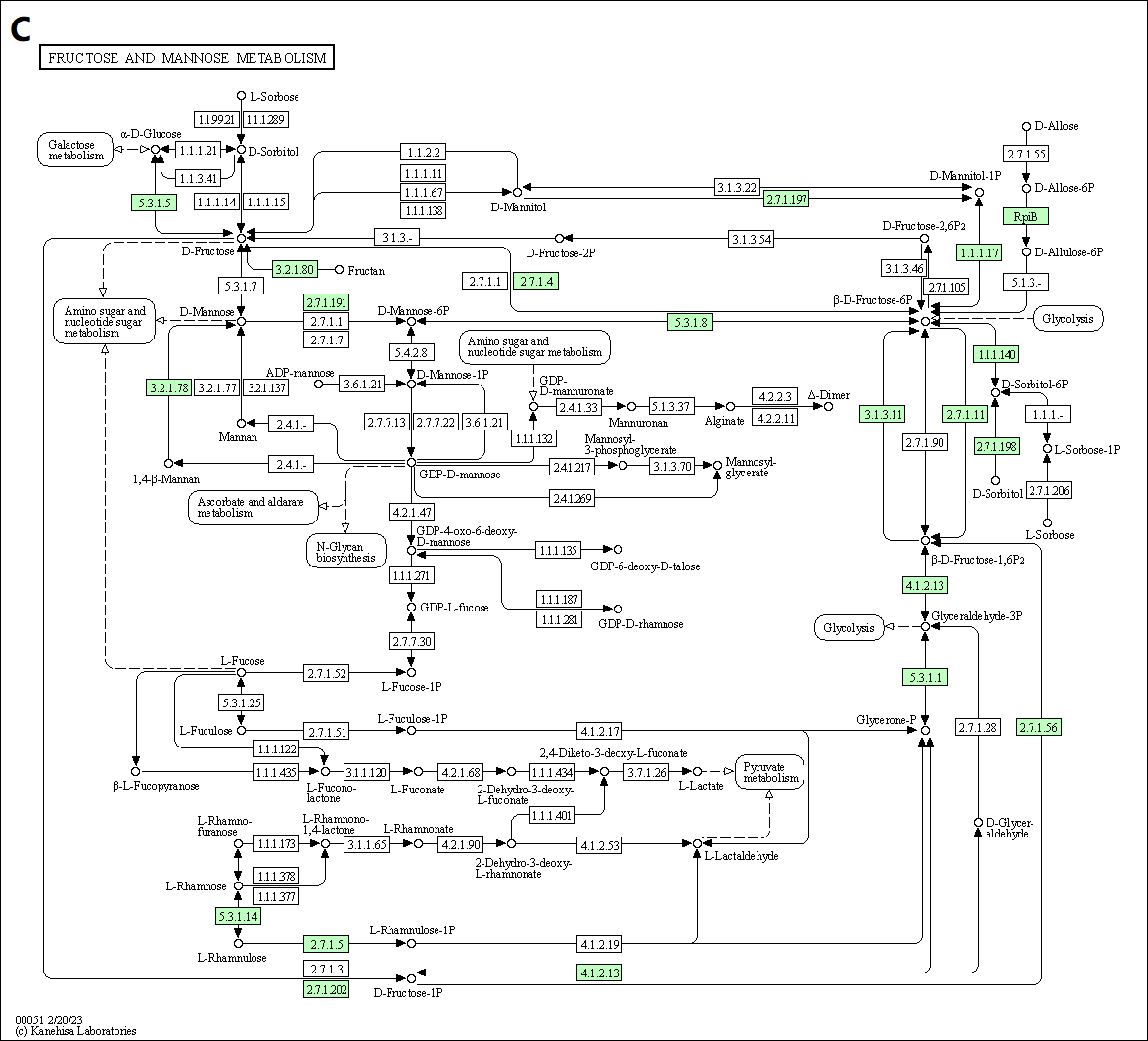


**Fig. S1.** Potential pathway of sugar metabolism process. (A) pentose phosphate pathway; (B) starch and sucrose metabolism; and (C) fructose and mannose metabolism. The green boxes represented the key genes contained in SR14.


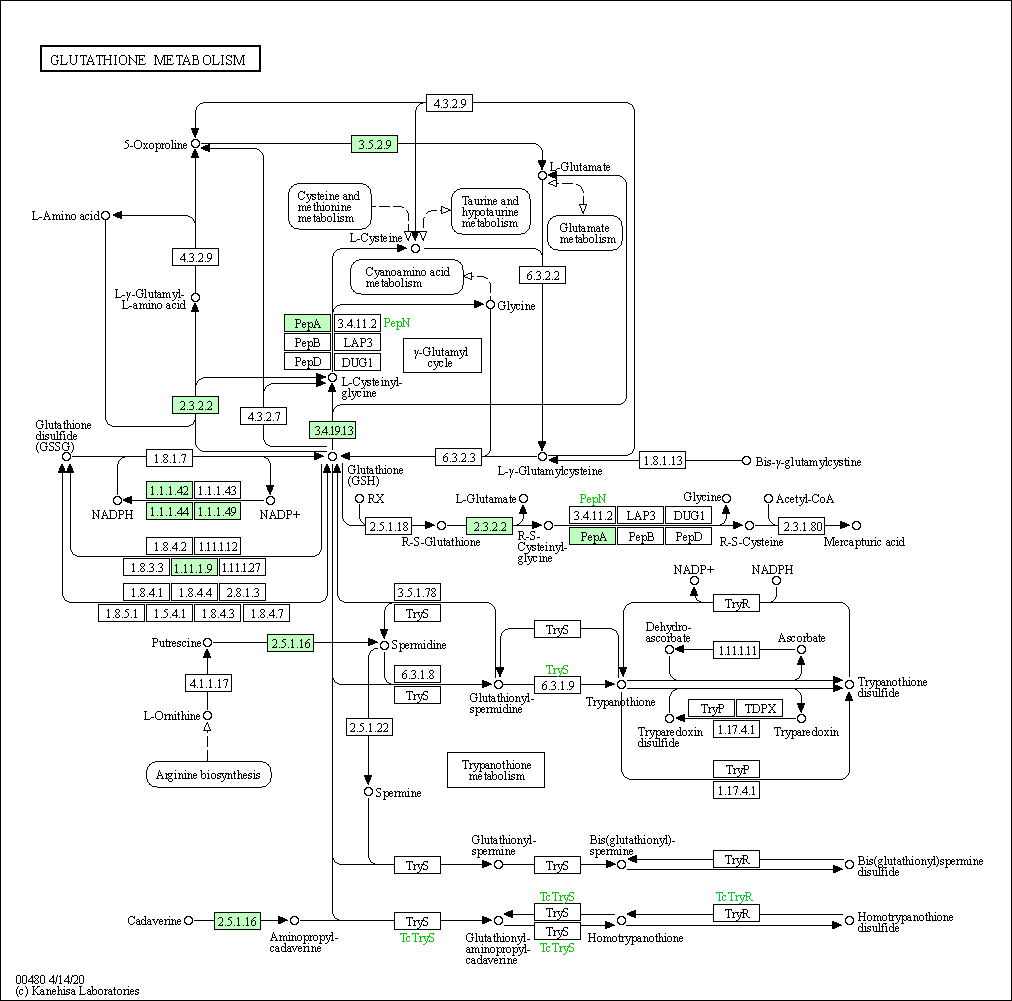


**Fig. S2.** Potential pathway of glutathione metabolism process. The green boxes represented the key genes contained in SR14.
